# Diversity of *Ooencyrtus* spp. (Hymenoptera: Encyrtidae) parasitizing the eggs of *Stenozygum coloratum* (Klug) (Hemiptera: Pentatomidae) with description of two new species

**DOI:** 10.1371/journal.pone.0205245

**Published:** 2018-11-07

**Authors:** Shahar Samra, Pasquale Cascone, John Noyes, Murad Ghanim, Alex Protasov, Emilio Guerrieri, Zvi Mendel

**Affiliations:** 1 Department of Entomology, the Volcani Center, Beit Dagan, Israel; 2 Faculty of Agriculture, the Hebrew University of Jerusalem, Rehovot, Israel; 3 Institute for Sustainable Plant Protection, the National Research Council of Italy, Portici (NA), Italy; 4 Department of Life Sciences, The Natural History Museum, London, United Kingdom; Sichuan University, CHINA

## Abstract

*Ooencyrtus* spp. (Hymenoptera, Chalcidoidea, Encyrtidae) are important natural enemies of agricultural and forest insect pests, and are distributed worldwide. Their reduced dimensions, highly variable morphological characters and possible effect of wide host range and abiotic factors, make correct identification at the species level particularly difficult. This paper combined molecular, morphological, and biological data to characterize a group of *Ooencyrtus* spp. emerging from the eggs of the variegated caper bug, *Stenozygum coloratum* in the east Mediterranean area. COI and ITS2 sequencing revealed the presence of six and five divergent clades, respectively. Three clades were identified as *Ooencyrtus telenomicida*, *Ooencyrtus pityocampae* and *O*. *pistaciae*. Two clades represent new species which are here described and named *Ooencyrtus zoeae and Ooencyrtus mevalbelus*. These features were combined with reliable morphological characters to facilitate the separation of these species. A dichotomous key and a new synonymy are proposed. *Ooencyrtus pistaciae* had two distinct COI clades but only one ITS2 clade. Crossbreeding trials that included *Ooencyrtus telenomicida*, *Ooencyrtus melvabelus sp*. *nov*. and *Ooencyrtus zoeae sp*. *nov*. confirmed their reproductive isolation. COI sequences showed 0–0.8% and 4–9% within and between-species genetic differences, respectively. ITS2 showed 0.4–5.9% genetic differences between species, with no genetic differences within species. Haplotype diversity of Israeli and Turkish populations of the various species was 0–0.98 and was particularly low in *Ooencyrtus pityocampae*, whose Israeli population showed no diversity. The discovery of the *Ooencyrtus* spp. on the eggs of the caper bug, and their abundance support the idea that the bug can be used as an alternative host for augmentation of populations of these parasitoids in agricultural and forestry systems.

## Introduction

In recent years molecular tools have been widely applied to facilitate the identification of important biocontrol agents [1] and to separate closely related parasitoid species [2–6]. The variation of morphological characters is often associated with host range, but abiotic factors may also play a significant role [7,8]. An example of the influence of temperature on the colour of adult parasitoids is given by *Meteorus pulchricornis* (Hymenoptera: Braconidae) [9].

The genus *Ooencyrtus* includes more than 300 known species, worldwide [10], and many of them are morphologically similar and often are difficult to distinguish [11,12]. Several *Ooencyrtus* species are considered important natural enemies of pest species [13–17]. However, the identification of species in this genus according to morphological features can be extremely difficult due to their small size and to the intraspecific variation of morphological characters generally used for identification of other congeners, e.g. leg colour, wing venation, antennal proportions and genitalia [10] [Noyes & Guerrieri pers. comm.].

Furthermore, there is a surprising lack of molecular information about species of this genus that could help in a correct identification at the species level.

*Ooencyrtus pityocampae* Mercet is a well-known egg parasitoid of the pine processionary moth (PPM) *Thaumetopoea pityocampae* Den and Schiff/*T*. *wilkinsoni* Tams species complex (Lepidoptera: Notodontidae), a major defoliator of pine throughout the Mediterranean basin [18–20]. This parasitoid is considered a generalist because it parasitizes eggs of a variety of species belonging to the Lepidoptera and the Heteroptera [21–24]. In the laboratory, *O*. *pityocampae* is easily reared on eggs of many other species of both insect orders [16,25–27]. In the east Mediterranean area it was recently identified in the eggs of the variegated caper bug (CB) *Stenozygum coloratum* (Klug) (Hemiptera: Pentatomidae) [28]. The CB is found in the Middle East and East Africa [29,30], and in Israel it is especially common on caper plants (*Capparis* spp.) growing within and on the edges of pine forests [31]. The CB oviposition period lasts throughout the spring and summer, mainly from May through September, and parasitism by *O*. *pityocampae* occurs throughout this period [31]. In Israel, the eggs of the PPM are found mainly in September through November [32], therefore it was assumed that *O*. *pityocampae* population alternated seasonally between these two hosts [30]. These data suggested that the CB plays a role in conservation of the *O*. *pityocampae* population, and potentially could be used for augmentation of OP populations in order to improve biological control of the PPM [30]. However, it soon became evident that the *Ooencyrtus* population on the CB eggs comprises of several additional closely related species, yet their identity was unclear. In an attempt to identify these species, preliminary molecular work was made. The analysis revealed the presence of several highly divergent genetic clades.

The present study aimed to obtain a clear picture of the identity of the *Ooencyrtus* species obtained from CB eggs by using an integrative approach, combining morphological, biological, and genetic data. We also compared the levels of genetic diversity of the various species. Finally, we examined the potential importance and use of this parasitoid guild for biological control, with emphasis on the PPM.

## Materials and methods

### Insect collection and rearing

CB egg clusters were sampled in 2010–2013 at various sites in Israel and southern Turkey (Table 1). No specific permissions were required for these locations/activities because they were not private properties or parks. Each egg cluster was placed in a separate glass tube (15mm wide and 100mm long) closed with cotton wool, and kept in the laboratory at 25°C, 40–60% RH and 14:10 L:D. Most emerging parasitoid individuals were transferred directly to 96% ethanol in 1.5-mL Eppendorf tubes and stored at -20°C, pending subsequent sampling for the molecular study. Some individual parasitoids (male/female pairs) from various sites were kept alive and reared in the laboratory, in order to obtain additional specimens for the crossbreeding experiments and for identification purposes (see below Section 2.3). Each female was placed inside a glass tube (sizes as above), together with a single male that emerged from the same egg cluster. To obtain maximum diversity, no more than one female/male pair was sampled from each egg cluster. For rearing, female parasitoids were given either CB or silk moth eggs (*Bombyx mori* L., Lepidoptera: Bombycidae) taken from our laboratory culture. A few offspring of each pair were transferred to 96% ethanol, as described above, and used for the molecular identification; a few others were preserved in 80% ethanol and used for morphological identification. In this way we ensured that the identity of each culture could be confirmed both morphologically and genetically. The identified lineages were used to create laboratory cultures of some of the species, which were used for the crossbreeding experiments.

**Table 1:** Information on *Ooencyrtus* spp. samples used in the present study.

Country	Site	Co-ordinates	Altitude (m)	Collection date	Collector*	Species**	No. of individuals
Israel	Gilboa	32°31'N; 35°22'E	170	4-Aug-10	SS	Om	1
Israel	Gilboa	32°31'N; 35°22'E	170	4-Aug-10	SS	Op	20
Israel	Yatir	31°20'N; 35°05'E	650	18-Aug-10	SS	Om	2
Israel	Yatir	31°20'N; 35°05'E	650	18-Aug-10	SS	Oz	10
Israel	Biriya	33°00'N; 35°29'E	780	30-Aug-10	SS	Ot	10
Israel	Haruvit	31°45'N; 34°55'E	135	4-Jun-11	SS	Op	14
Israel	Lahav	31°22'N; 34°51'E	480	19-Jul-11	SS	Om	7
Israel	Lahav	31°22'N; 34°51'E	480	19-Jul-11	SS	Op	15
Israel	Eshta'ol	31°48'N; 35°00'E	260	26-Jul-11	SS	Op	3
Israel	Rosh Pinna	32°57'N; 35°32'E	380	12-Aug-11	GDC	Onb	3
Israel	Rosh Pinna	32°57'N; 35°32'E	380	12-Aug-11	GDC	Om	2
Israel	Rosh Pinna	32°57'N; 35°32'E	380	12-Aug-11	GDC	Oz	1
Israel	Rosh Pinna	32°57'N; 35°32'E	380	12-Aug-11	GDC	Ot	10
Israel	Rosh Pinna	32°57'N; 35°32'E	380	12-Aug-11	GDC	Op	3
Israel	Eyn-Gev	32°79'N; 35°64'E	-200	20-Aug-11	GDC	Ona	1
Israel	Gilboa	32°31'N; 35°22'E	170	15-Aug-12	SS	Om	9
Israel	Gilboa	32°31'N; 35°22'E	170	15-Aug-12	SS	Ot	17
Israel	Avdat	30°79'N; 34°77'E	540	24-Jun-13	SS	Ona	4
Israel	Avdat	30°79'N; 34°77'E	540	24-Jun-13	SS	Oz	2
Turkey	Antakya	36°12'N; 36°10'E	140	5-Sep-10	MD	Oz	1
Turkey	Karataş	36°33'N; 35°32'E	25	23-Jun-13	FCC	Op	18
Turkey	Antakya	36°12'N; 36°10'E	140	26-Jul-13	SS	Oz	1
Turkey	Antakya	36°12'N; 36°10'E	140	26-Jul-13	SS	Ot	53
Turkey	Antakya	36°12'N; 36°10'E	140	26-Jul-13	SS	Op	36
Turkey	Tarsus	36°57'N; 34°54'E	50	27-Jul-13	SS	Oz	26
Turkey	Tarsus	36°57'N; 34°54'E	50	27-Jul-13	SS	Op	5
USA	Saint Mary's Co., Maryland	38° 6'N; 76°21'W	5	3-Aug-11	BT	Ok	1

* Collectors: SS = Shahar Samra; CJC = Carlos Jorge Carvalho; MD = Miktat Doğanlar; FCC = Feza Can Cengiz; BT–Bob Tatman.

** Ona/Onb–*O*. *pistaciae* types a and b; Om–*O*. *mevalbelus*; Oz = *O*. zoeae; Ot = *O*. *telenomicida*; Op = *O*. *pityocampae*; Ok = *O*. *kuvanae*

### Molecular characterization

A total of 274 *Ooencyrtus* individuals were sampled for the genetic analysis; all had emerged from CB eggs collected at various sites in Israel and southern Turkey (Table 1). Additionally, one *O*. *kuvanae* individual which emerged from the eggs of *Lymantria dispar* collected in Maryland, USA, served as an outgroup in the analysis. Total genomic DNA was extracted from each individual wasp with the Qiagen DNeasy Blood and Tissue kit (Qiagen, Redwood City, CA, USA) according to the manufacturer's protocol. Two DNA fragments were analyzed: a 946-bp fragment of the mitochondrial Cytochrom Oxidase 1 (COI) gene, and an 889–933-bp nuclear DNA segment that comprised a partial (65 bp) fragment of the ribosomal 5.8S unit, a complete (481–525 bp) sequence of the ribosomal Internal Transcribed Spacer 2 (ITS2), and a partial (342 bp) fragment of the 28S ribosomal unit.

The initial primer sequences used for COI were kindly provided by Marie-Anne Auger-Rozenberg) Unité de Zoologie Forestière, INRA, Orléans, France):

forward– 5' CGAATAAATAATATAAGTTTTTG 3' and
reverse– 5' CAACATAAATAAGAATCTGGA 3').

However, the forward primer often attached to the middle of the COI segment, so that only the second half of the segment was obtained. To solve this problem, another forward primer was designed, based on the previous one:

5'CTCGAATAAATAATATAAGATTTTG3'.

We designed the primers for ITS2 sequences, based on an alignment of ITS2 sequences of Chalcidoidea superfamily members, which were available in NCBI. The forward and reverse primers were, respectively, 5' GAACTGCAGGACACATGAACA 3' and 5' CTTGTTCGCTATCGGTCTCGTGGT 3'.

Amplification of the COI and ITS2 used the following conditions: Initial denaturation at 95°C for 3 min, followed by 36 cycles of 95°C for 30 s, 54°C for 30 s, and 72°C for 75 s (COI) or 1 min (ITS2), followed by final extension at 72°C for 5 min.

The PCR for COI was performed in a 30-μL reaction volume containing a 3-μL sample, 0.3 μL (30 pmole) of each primer, 0.6 μL of dNTPs (10 mM of each), 0.15 μL (0.75 units) of Taq polymerase (Fermentas, Vilnius, Lithuania), 0.6 μL of MgCl_2_ (to a final concentration of 2.5 mM) and 22.05 μL of dd H_2_O. The PCR for ITS2 was performed with the same reagent concentrations except for MgCl_2_, which was at a final concentration of 2 mM.

To ensure good-quality sequencing results, PCR products were electrophoresed through 1% Agarose gel, and the DNA segment was cut from the gel and later extracted and purified with the RBC-YDF HiYield Gel/PCR DNA Fragments Extraction Kit (RBC, Banqiao City, Taiwan). Sequencing was done by Macrogen Corp. (Seoul, S. Korea). The PCR products of each individual, obtained with the reverse and forward primers, were aligned and compared by means of the ChromasPro software, Version 1.6 (Technelysium Pty Ltd, South Brisbane, Queensland, Australia), and were checked manually for errors.

The COI sequences were aligned manually and were translated into amino acids in order to verify that none of the sequences contained any stop codon, which could indicate the presence of numts. There were no gaps in the COI alignment. The ITS2 sequences were aligned by means of the Mafft (ver. 7) Q-ins-I method [33]. Additionally, the COI sequence of *Nasonia longicornis* Darling (Hymenoptera: Pteromalidae) (Accession No. EU746612) was downloaded from GeneBank and used as a second outgroup (together with *O*. *kuvanae* Howard) in the COI phylogenetic analysis.

The Mega program (ver. 6) was used to calculate genetic distances within and between species, according to the Kimura 2-parameter model[34]; the same program was used to construct Maximum Likelihood phylogenetic trees of COI and ITS2 sequences, with bootstrap support (1000 replicates). The DNASP v5.10.1 program [35] and Arlequin v.3.5.1.3 software [36] were used to compute haplotype and nucleotide diversity indices.

Nucleotide sequences have been deposited in GeneBank under accession numbers KP676603- KP676668, KM485909, KM485911–KM485915 (COI), KP676669–KP676674 and KM527071–KM527072 (ITS2).

### Morphological characterization

Live parasitoids were killed in ethanol 80% and kept at –20°C until preparation. All parasitoids were critically point dried [37] and mounted on card for further examination. Selected card mounted specimens were slide mounted following Noyes [37].

### Abbreviations used

F1-F2, F.…: first funicular segment, second funicular segment etc; FV, minimum frontovertex width; FWL, maximum fore wing length; FWW, maximum fore wing width; GL, gonostylus (third valvula) length; HW, maximum head width; MS, the shortest length of malar space; MT, mid-tibia length; MV, marginal vein; OCL, occipital–ocellar line (the shortest distance between posterior ocellus and occipital margin); OD, maximum diameter of posterior ocellus; OL, ovipositor length; OOL, ocular–ocellar line (the shortest distance between posterior ocellus and adjacent eye margin); PMV, post marginal vein; POL, the shortest distance between posterior ocelli; SL, scape length (excluding radicle); SMV, submarginal vein, SV, stigmal vein; SW, maximum scape width. Depositories: BMNH—Department of Life Sciences, Natural History Museum, London, England, UK; DEZA- former Department of Entomology and Zoology (now: Laboratory of Entomology Ermenegildo Tremblay), University of Naples Federico II, Portici, Naples, Italy; IARI—National Pusa Collection, Division of Entomology, Indian Agricultural Research Institute, New Delhi, India; MHNG—Musée d' Histoire Naturelle, Geneva, Switzerland; MNCN—Museo Nacional de Ciencias Naturales, Madrid, Spain; TAUI—The Steinhardt Museum of Natural History, Tel Aviv- Tel Aviv University Insect Collection, Tel Aviv, Israel.

Unless otherwise specified all data on distribution and hosts of *Ooencyrtus* are from Universal Chalcidoidea Database [10].

### Crossbreeding trials

This experiment was conducted to determine whether interspecific breeding between the studied species could occur. We tested the three most similar morphospecies obtained in the present study, which later were identified as *O*. *telenomicida*, *O*. *mevalbelus* and *O*. *zoeae*, of which the second and third were also the most closely related. Glass-tube arenas, each containing three virgin females of one species and two males of another species, were set up in the laboratory, at 25°C,40–60% RH and 14:10 L:D. These individuals were provided with honey-water solution on a piece of cloth, and left for 3 days before adding 100 silk moth eggs. After 3–5 days adult parasitoids were removed and eggs were kept at the same laboratory conditions till the emergence of new adults. Because the three studied species reproduce sexually (Shahar unpubl. observations), females are expected among the offspring only in case of successful crossbreeding. Five repetitions were made for each crossbreeding treatment, e.g., ♀♀ species1 with ♂♂ species 2, etc. Females and males of the same species were placed together under similar conditions served as controls.

### Nomenclatural acts

The electronic edition of this article conforms to the requirements of the amended International Code of Zoological Nomenclature, and hence the new names contained herein are available under that Code from the electronic edition of this article. This published work and the nomenclatural acts it contains have been registered in ZooBank, the online registration system for the ICZN. The ZooBank LSIDs (Life Science Identifiers) can be resolved and the associated information viewed through any standard web browser by appending the LSID to the prefix "http://zoobank.org/". The LSID for this publication is: *urn*:*lsid*:*zoobank*.*org*:*pub*:*1B2BAB83-8146-4AE3-A28D-EFF873083A12*. The electronic edition of this work was published in a journal with an ISSN, and has been archived and is available from the following digital repositories: PubMed Central, LOCKSS.

## Results

### Phylogenetic analysis

**Fig 1:**
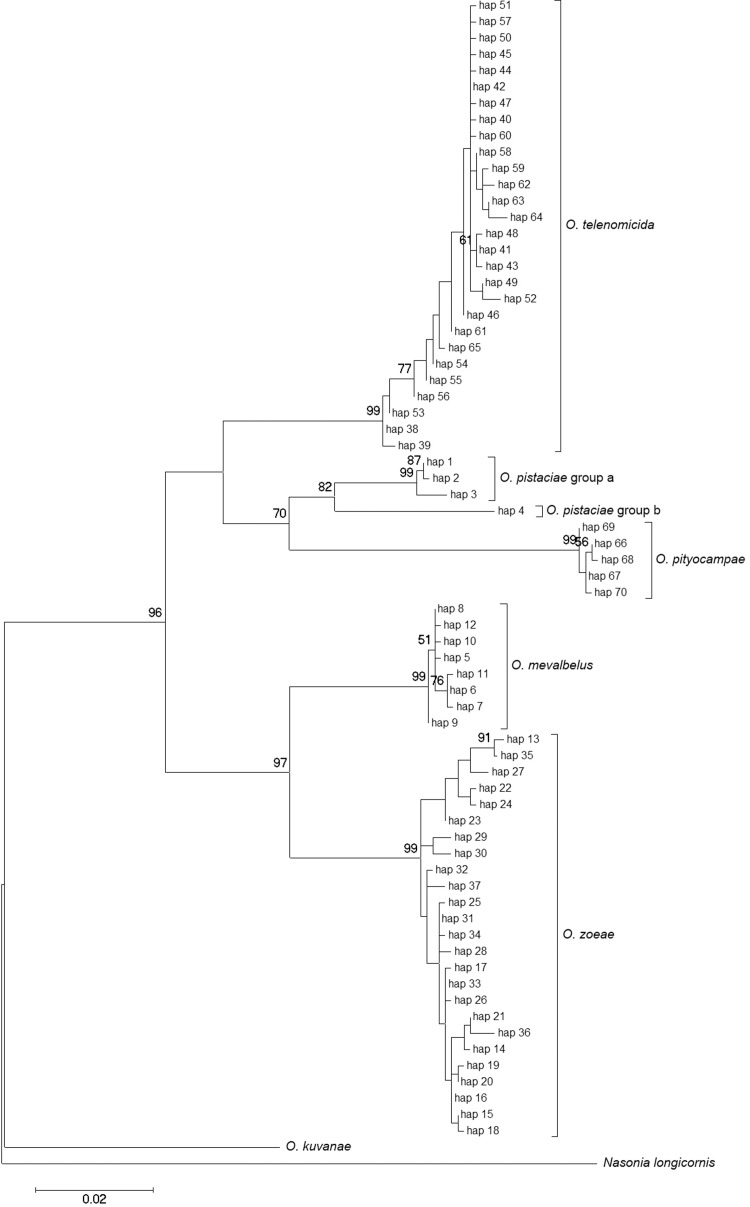
Maximum likelihood tree for COI haplotypes, using Kimura 2-parameter model distances. Bootstrap values greater than 50% are shown.

**Fig 2:**
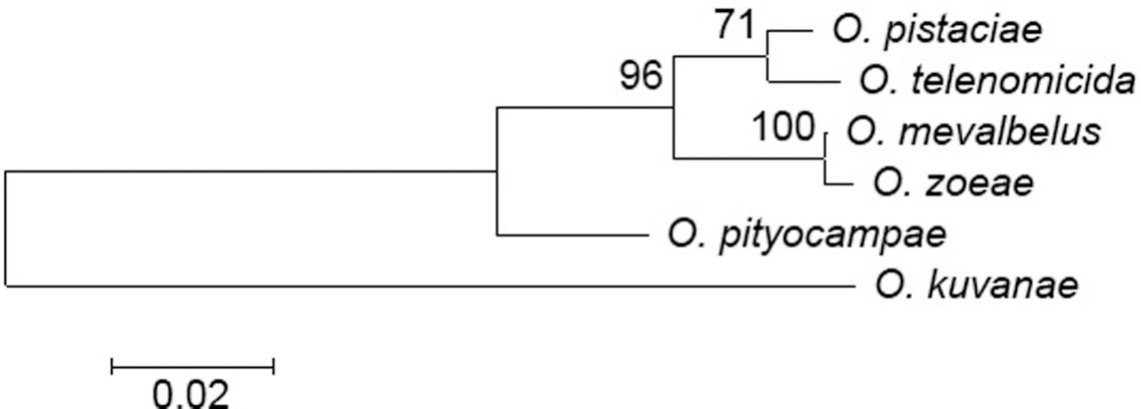
Maximum likelihood tree for ITS2 haplotypes, using Kimura 2-parameter model distances. Bootstrap values greater than 50% are shown.

In general, molecular analysis revealed the presence of six (COI, Fig 1) or five (ITS2, Fig 2) genetically distinct clades. The partition of individuals into groups was generally similar for both DNA fragments, although there were some exceptions (described below in this Section). Three of these groups belonged to known species, identified as *O*. *pistaciae*, *O*. *telenomicida* and *O*. *pityocampae*. Two groups of morphologically similar individuals had two distinct COI clades, but identical ITS2 sequences, and both are here identified as *O*. *pistaciae*. The other two species presently identified, are the most closely related, both morphologically and genetically, and were named *O*. *zoeae*. and O. *mevalbelus*). All species and groups were found in Israel, but only *O*. *zoeae*, *O*. *telenomicida* and *O*. *pityocampae* were also found in Turkey.

The COI mean genetic distances within species ranged between 0.000 and 0.008, i.e., differences of 0–0.8% (Table 2); and genetic distances between species ranged between 0.044 and 0.090 (Table 3). The most closely related were *O*. *pistaciae* groups a and b, separated by a mean genetic distance of 0.044, and, as mentioned above, they are suspected of belonging to the same species. The next most closely related pair were *O*. *mevalbelus* and *O*. *zoeae*, separated by 0.050. The greatest COI mean genetic distance—of 0.090—was obtained between *O*. *pityocampae* and the pair *O*. *mevalbelus* and *O*. *zoeae*. Genetic distances of ITS2 sequences were generally lower: 0.004–0.059 between species, with no observed genetic variability within species (Table 4). Furthermore, the two *O*. *pistaciae* COI clades had identical ITS2 sequences. Slightly different relationships were obtained with the ITS2 sequences from those obtained with the COI ones. According to the ITS2 analysis, the most prominent difference was assigned to *O*. *pityocampae*. The latter species was the most distinct from all other examined congeners; with genetic distances of 0.052–0.059, compared with 0.004–0.044 between all the others (Table 3). Whereas according to the COI sequences, *O*. *pityocampae* was more closely related to *O*. *telenomicida* and *O*. *pistaciae*, with separations of 0.052 and 0.055, respectively, than *O*. *mevalbelus* and *O*. *zoeae*, with separations of 0.073 and 0.083, respectively. Therefore, the last two were placed separately from the other three species in the COI phylogenetic analysis (Fig 1). *O*. *pityocampae*, *O*. *telenomicida* and *O*. *pistaciae* form a monophyletic group with 96% bootstrap, thus supporting their separation from the other two species, whereas according to the ITS2 phylogeny *O*. *pityocampae* was placed outside the monophyletic group of the other four species. In both analyses *O*. *mevalbelus* and *O*. *zoeae* formed a monophyletic group, with 96% bootstrap support.

**Table 2:** Overall mean distance (±SE) over COI sequence pairs within species groups, determined by using the Kimura 2-parameter model.

	Kimura2 distance	SE
*O*. *pistaciae* a	0.003	0.001
*O*. *pistaciae* b	0.000	0.000
*O*. *mevalbelus*	0.002	0.001
*O*. *zoeae*	0.008	0.002
*O*. *telenomicida*	0.005	0.001
*O*. *pityocampae*	0.001	0.001

**Table 3:** Overall mean distance over COI sequence pairs between species groups, as determined by using the Kimura 2-parameter model.

	1	2	3	4	5	6
1. *O*. *pistaciae* a		0.006	0.009	0.009	0.008	0.008
2. *O*. *pistaciae* b	0.044		0.009	0.009	0.009	0.009
3. *O*. *mevalbelus*	0.077	0.080		0.007	0.009	0.009
4. *O*. *zoeae*	0.081	0.083	0.050		0.009	0.009
5. *O*. *telenomicida*	0.065	0.073	0.081	0.078		0.010
6. *O*. *pityocampae*	0.068	0.076	0.090	0.090	0.086	

Distances and standard errors are given below and above the diagonal, respectively.

**Table 4:** Mean distance over ITS2 sequence pairs between species groups, as determined by using the Kimura 2-parameter model.

	1	2	3	4
1. *O*. *pistaciae* a and b				
2. *O*. *mevalbelus*	0.033			
3. *O*. *zoeae*	0.038	0.004		
4. *O*. *telenomicida*	0.015	0.039	0.044	
5. *O*. *pityocampae*	0.055	0.057	0.059	0.052

### Genetic diversity analysis

A total of 70 COI haplotypes were obtained: 1–15 for each population (species and country; Table 5). Haplotype diversity ranged between 0 and 0.955, nucleotide diversity was 0–0.037, and Өs values were 0–6.289 for all populations of species from Turkey and Israel. *O*. *pityocampae* was the least diverse, with lower haplotype diversity (0.610) than all other species—in which it ranged from 0.800 to 0.955—apart from *O*. *pistaciae* group b, of which only three individuals, with identical haplotype, were obtained. This was even more conspicuous among the Israeli *O*. *pityocampae* population, in which, in spite of the relatively large sample, no genetic diversity was found in the COI sequences, with only a single haplotype obtained, compared with five haplotypes in the Turkish population, and 3–15 haplotypes in the populations of all the other species from either Israel or Turkey.

**Table 5:** Indices of genetic diversity for COI sequences of *Ooencyrtus* spp. populations obtained from *S*. *coloratum* eggs collected in Israel and Turkey.

Species	Country	Number of individuals	Number of haplotypes	Haplotype diversity *H* (±SD)	Nucleotide diversity, π (±SD)	Өs (per sequence) (±SD)*
*O*. *pistaciae* a	Israel	5	3	0.800 ± 0.02	0.013 ± 0.01	3.360 ± 2.00
*O*. *pistaciae* b	Israel	3	1	0.000 ± 0.00	0.000 ± 0.00	0.000 ± 0.00
*O*. *mevalbelus*	Israel	21	8	0.840 ± 0.00	0.002 ± 0.00	2.224 ± 1.09
*O*. *zoeae*	Israel	13	12	0.980 ± 0.04	0.030 ± 0.02	5.800 ± 2.51
	Turkey	26	14	0.930 ± 0.03	0.037 ± 0.02	6.289 ± 2.32
	Total	39	25	0.955 ± 0.02	0.035 ± 0.02	6.150 ± 2.12
*O*. *telenomicida*	Israel	39	15	0.924 ± 0.01	0.013 ± 0.01	4.730 ± 1.70
	Turkey	53	14	0.898 ± 0.02	0.019 ± 0.01	3.746 ± 1.40
	Total	92	28	0.926 ± 0.01	0.019 ± 0.01	5.301 ± 1.66
*O*. *pityocampae*	Israel	55	1	0.000 ± 0.00	0.000 ± 0.00	0.000 ± 0.00
	Turkey	59	5	0.529 ± 0.07	0.004 ± 0.00	0.861 ± 0.47
	Total	114	5	0.610 ± 0.03	0.003 ± 0.00	0.753 ± 0.41
Total	-	274	70	0.923 ± 0.01	0.058 ± 0.00	25.370 ± 5.51

* Өs = estimated population effective size multiplied by the mutation rate.

### Crossbreeding trials

All interspecies crossings between *O*. *mevalbelus*, *O*. *zoeae*, and *O*. *telenomicida* resulted in male offspring only, whereas all control (within-species crossings) trials resulted in both male and female offspring (29–62% females in all replicates). In conclusion, successful cross-mating and hybridization between these three species did not occur.

### Morphological identification

**Table:** 

Key to ♀♀		
1	Legs with at least hind coxa partially marked brown	2
-	Legs, including all coxae, completely pale orange	3
2 (1)	Mesoscutum and scutellum metallic dark green	*pityocampae*
-	Mesoscutum and scutellum mostly coppery	*pistaciae*
3 (1)	Head mostly bright metallic dark green (especially gena and lower face)	*mevalbelus* sp. nov.
-	Head generally dull, mostly dull coppery purple with a little dull, dark green	4
4 (3)	Mesoscutum with a moderate coppery purple sheen, sides anteriorly weakly metallic dark green; larger specimens (>1mm) with F1 at least as long as F2 (higher half of interantennal prominence almost dull; sculpture on middle scutellum rounded)	*zoeae* sp. nov.
-	Mesoscutum with a slightly brassy, moderate metallic dark green sheen, a little coppery purple anteriorly and along posterior margin; F1 always shorter than F2 (higher half of interantennal prominence purple shiny; sculpture on middle scutellum striate/elongate)	*telenomicida*

**Fig 3:**
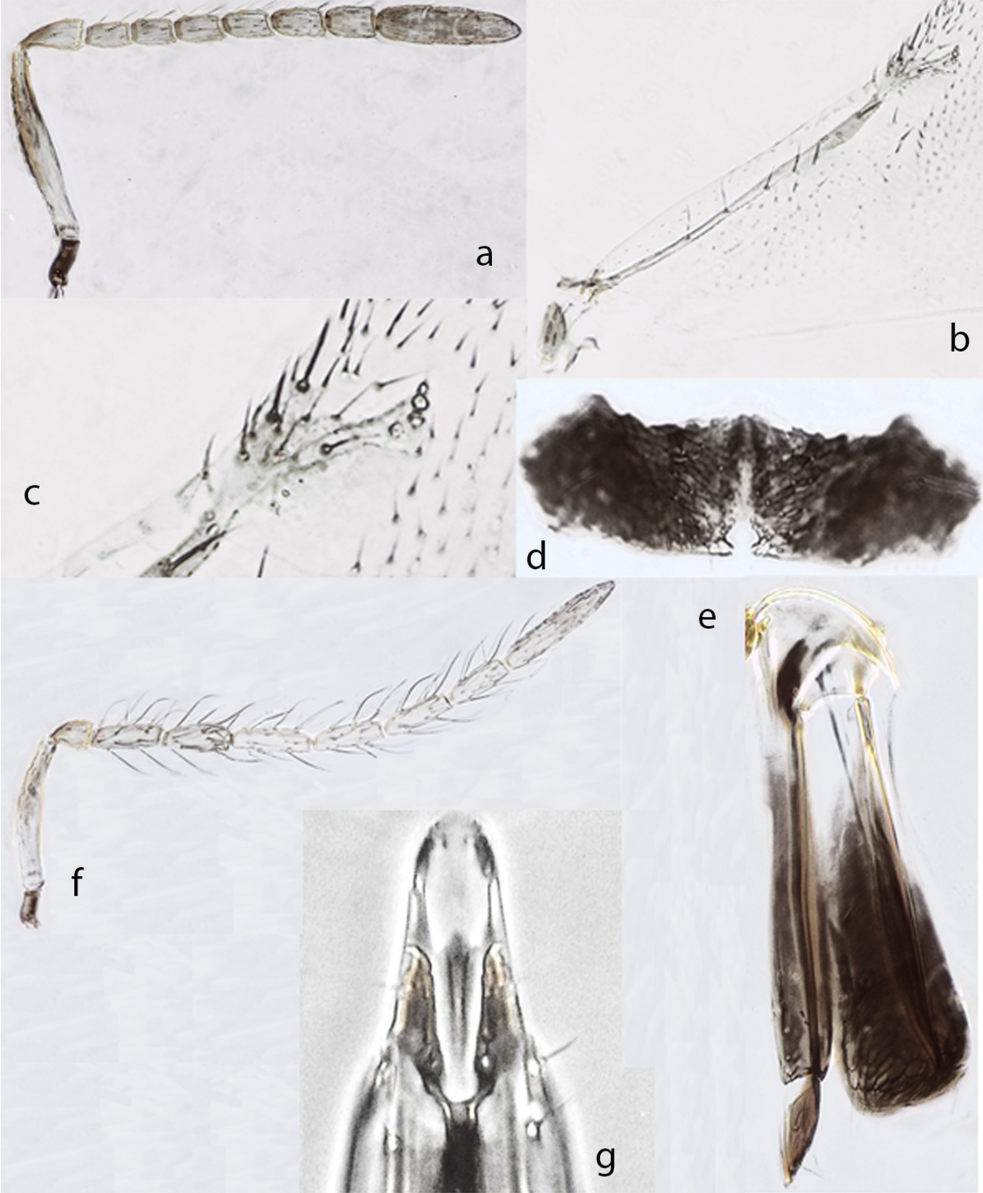
*Ooencyrtus zoeae* Guerrieri & Samra sp. nov.♀. a. Antenna. b. Fore wing. c. Fore wing venation. d. Hypopygium. e. Ovipositor. ♂. f. Antenna. g. Genitalia.

*Ooencyrtus zoeae* Guerrieri & Samra sp. nov. *urn*:*lsid*:*zoobank*.*org*:*act*:*603C33D5-ECB3-4809-B500-DB60C64C786E* (Fig 3)

Female. Holotype, length 0.9mm. Head black with very faint metallic lustre on frontovertex, interantennal prominence with green-blue reflections; mesothorax slightly shiny, scutellum dull with a faint green lustre at apex; antennal scape (Fig 3A) yellow with a narrow brown stripe along dorsal margin (except the basal third), remaining parts of antenna brown except the apical half of pedicel appearing paler brown; thorax black, setae on dorsum of thorax slightly silvery; tegula black; legs yellow except apices of all tarsi, brown; wings hyaline, venation brown; gaster black, the first 2 segments yellowish.

Head on frontovertex with fine, shallow, fairly regular, polygonally reticulate sculpture of mesh size clearly less smaller than an eye facet; ocellar angle about 75°; occipital margin sharp but not carinate; head about 3.7× as broad as frontovertex; antenna (Fig 3A) with scape about 6× as long as broad; all funicular segments longer than broad and with linear sensilla; clava basal suture perpendicular, apical one oblique and with a sensory area along ventral surface of the last segment so that giving it has a slightly obliquely truncate appearance; mandible with 2 teeth and a broad truncation; mesoscutum with sculpture shallow, made of polygonal irregular cells; scutellum with sculpture coarse, made of elongate cells at sides and rounded in the middle; fore wing (Fig 3B) about twice as long as broad with marginal vein (Fig 3C) 1.5× as long as broad, postmarginal vein punctiform; mid tibial spur shorter than mid basitarsus; gaster with hypopygium extending not more than 0.6× the gaster length; ovipositor hardly exserted. Relative measurements: HW 27, FVW 7.5, POL 3, OOL <1, OCL 4, OD 1, SL 14, SW 2.5, FWL 64, FWW 30, SMV 24, MV 2, PMV 1, SV 3.

Paratype: Hypopygium (Fig 3D) very transverse about 3× as broad as long, its posterior margin with a median invagination; ovipositor (Fig 3E) about as long as mid tibia or about 5× as long as gonostylus. Gonostylus (Fig 3E) about 0.7× as long as mid tibial spur; second valvifer with 4 or 5 subapical setae. Relative measurements: OL 76, MT 77, GL 14

Variation. Virtually none in the material at hand except for the body length, which varies from 0.8 to 1.1mm.

Male (length 0.8mm): generally similar in appearance to female except for antenna (Fig 3F) and genitalia (Fig 3G); antenna (Fig 3F) with all funicular segments much longer than broad and clothed in setae that are generally much longer than diameter of segments; genitalia (Fig 3G) without parameres and with a single pair of setae at apex; digitus about 3.5× as long as broad and with 1 apical hook; aedeagus slender, apically rounded and about 0.6× as long as mid tibia.

Hosts. Reared from eggs of *Stenozygum coloratum* (Klug) (Hemiptera: Pentatomidae) on caper (*Capparis spinosa* L).

Distribution. Israel, Turkey

Material examined. Type material. Holotype ♀, ISRAEL Bet Dagan laboratory colony ex eggs of silk moth 2.ii.2015 (Shahar Samra) (initial strain collected at Israel Ein-Avdat 30°49'N; 34°45'E, 500m asl, 24.vi.2013 ex *Stenozygum coloratum* on caper—Shahar Samra) (TAUI). Paratypes: 15♀, 12♂ same data as holotype (TAUI, BMNH, DEZA). Non-type material: ISRAEL, 3♀, 7♂, Avdat, 30°49'N 34°45'E, 500m, 24.vi.2013, Orig. field reared ex eggs *Stenozygium coloratum*, Lab reared ex eggs *Bombyx mori* (S. Samra #8); 7♀, 6♂, Ein-Avdat, 30°49'N 34°45'E, 490m, 5.vi.2013, Field/Lab. reared ex eggs *Stenozygium coloratum*, Lab reared ex eggs *Bombyx mori* (S. Samra #2); TURKEY, 1♀, Antakya 36°12'N; 36°10'E 140m, 5.ix.2010, Field/Lab. reared ex eggs *Stenozygium coloratum*, Lab reared ex eggs *Bombyx mori* (Miktat Doğanlar); TURKEY, 10♀, 4♂, Antakya 36°12'N; 36°10'E 140m, 26.vii.2013, Field/Lab. reared ex eggs *Stenozygium coloratum*, Lab reared ex eggs *Bombyx mori* (S. Samra)

Comments: The new species is virtually undistinguishable from *O*. *mevalbelus* with which shares a number of morphological features including relative widths of frontovertex, scape, wing and ovipositor proportions. The small differences noted in the key could generally be considered to fall within intraspecific variation but molecular and biological data confirm the different identity of this species. The species is named in honour of Zoe Elizabeth Good.

**Fig 4:**
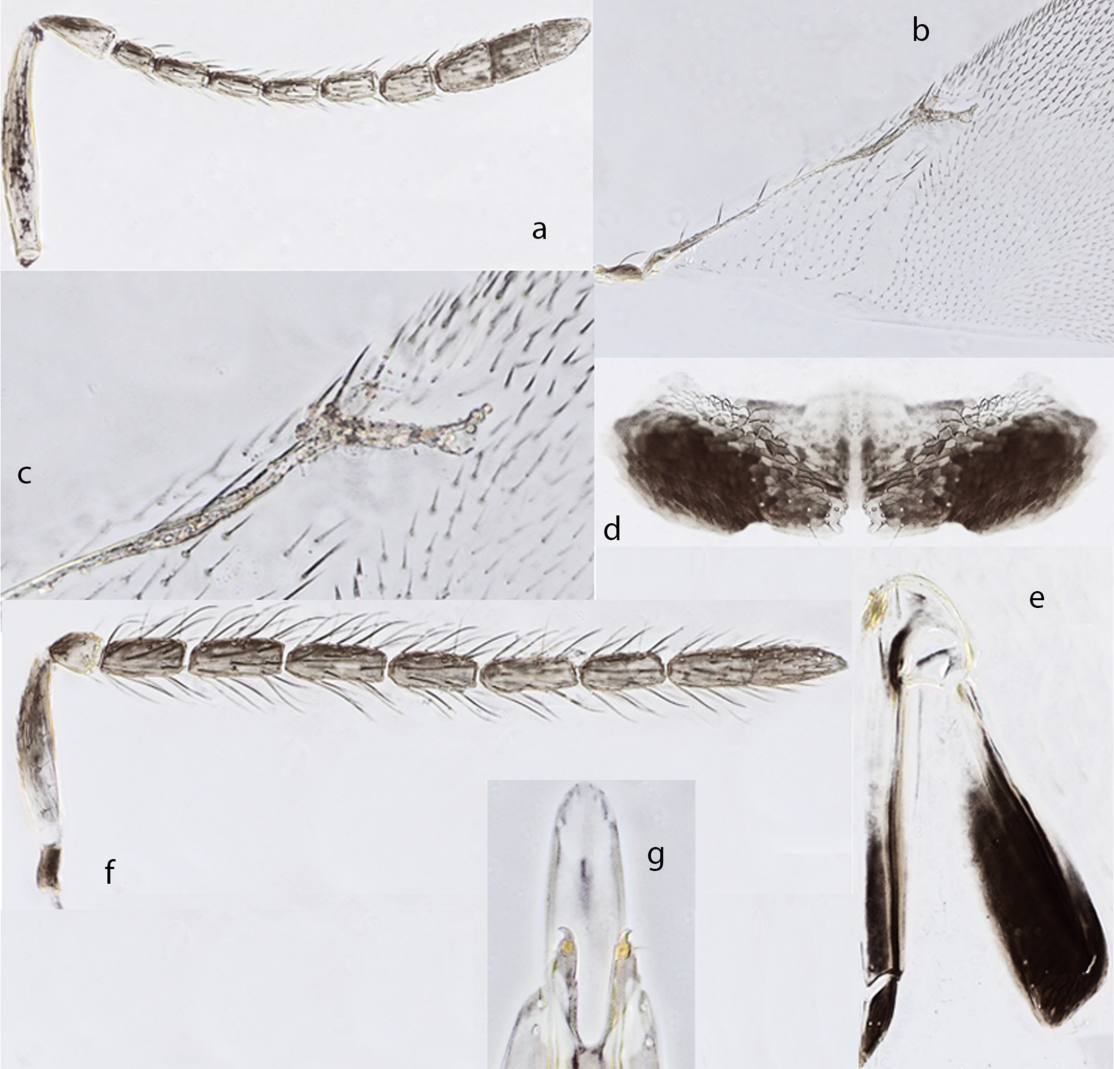
*Ooencyrtus telenomicida* (Vassiljev) ♀. a. Antenna. b. Fore wing. c. Fore wing venation. d. Hypopygium. e. Ovipositor. ♂. f. Antenna. g. Genitalia.

*Ooencyrtus telenomicida* (Vassiljev) (Fig 4)

*Encyrtus telenomicida* Vassiliev, 1904: 104. Type material Urkaine. Probably lost. Not examined.

*Schedius flavofasciatus* Mercet, 1921: 315. Lectotype ♀, designated Noyes, 1981:182, MNCN, examined. Synonymy with *telenomicida* by Ferriére & Voegelé, 1961: 32.

*Schedius telenomicida* (Vassiliev); Meyer, 1943:35–36.

*Ooencyrtus telenomicida* (Vassiliev); Romanova, 1953:238,240,246.

*Ooencyrtus flavofasciatus* (Mercet); Herting, 1976:98–99.

Diagnosis. Female—Head black with a very faint sheen; interantennal prominence with blue-purplish reflections; antenna (Fig 4A) brown except scape yellow with a brown stripe along dorsal margin and apical half of pedicel yellow; thorax black with a very faint dark green sheen on mesoscutum; tegula black; mesopleuron black with a faint purple sheen; legs yellow, tarsi a little darker; wings hyaline, venation brown, marginal vein appearing darker; basal half of gaster yellow (up to third tergite), remaining part brown; gonostylus black.

Head about 4× as wide as frontovertex; ocellar angle about 60°; antenna (Fig 4A) with scape slender, 7.4× as long as broad; all funicular segments distinctly longer than broad, F2–F6 with linear sensilla, clava slender, pointed at apex, sensory area at apex only; mandible with 1 tooth and a broad truncation. Thorax with fine, polygonally reticulate sculpture on mesoscutum that is shallower than that on frontovertex; scutellum with minute and longitudinally elongate sculpture that is deeper than that on mesoscutum and giving a striate appearance; setae on mesoscutum thin and longer than those on scutellum; scutellum in profile virtually flat; mesopleuron overreaching the base of gaster; fore wing about (Fig 4B) 2.4× as long as broad, with marginal vein (Fig 4C) about 1× as long as broad, postmarginal vein rudimental.

Hypopygium (Fig 4D) reaching two/third from apex of gaster with posterior margin slightly produced medially; ovipositor (Fig 4E) hardly exserted, about 0.9× as long as mid tibia and 5× gonostylus, gonostylus shorter than mid tibial spur. Relative measurements: HW 28, FVW 7, POL 4, OOL <1, OCL 2, OD 1, SL 15, SW 2, FWL 60, FWW 24, SMV 22, MV 1.5, PMV<1, SV 3; OL 72, MTL 80, GL 15

Male. Similar to female except for antenna (Fig 4F) and genitalia (Fig 4G).

Hosts. *Stenozygium coloratum*; *Aelia sp*., *A*. *acuminata*, *A*. *cognata*, *A*. *germari*, *A*. *rostrata*, *Carpocoris fuscispina*, *C*. *pudica* (Hemiptera: Pentatmidae); *Coreus marginatus*; *Coriomeris denticulatus* (Hemiptera: Coreidae); *Dolycoris sp*., *D*. *baccarum*, *D*. *numidicus*, *D*. *penicillata* (Hemiptera: Pentatomidae); *Enoplops cornutus* (Hemiptera: Coreidae); *Euproctis chrysorrhoea* (Lepidoptera: Erebidae); *Eurydema ornatum* (Hemiptera: Pentatomidae); *Eurygaster sp*., *E*. *austriaca*, *E*. *integriceps*, *E*. *maura* (Hemiptera: Scutellaridae); *Graphosoma sp*., *G*. *lineatum*, *G*. *semipunctatum* (Hemiptera: Pentatomidae); *Laothoe populi* (Lepidoptera: Sphingidae); *Nezara viridula*; *Palomena sp*., *P*. *prasina* (Hemiptera: Pentatomidae); *Pyrrhocoris apterus* (Hemiptera: Pyrrhocoridae); *Rhynocoris iracundus* (Hemiptera: Reduviidae); *Solenosthedium lynceum* (Hemiptera: Scutellaridae); *Streblote repanda* (Lepidoptera: Lasiocampidae); *Eurydema integriceps* (Hemiptera: Pentatomidae)[38]; *Acrosternum arabicum*, *A*.*breviceps*; *Brachynema germari* (Hemiptera: Pentatomidae) on *Pistacia vera* L. (Anacardiaceae) [39].

Distribution. Israel; Armenia; Azerbaijan; Czech Republic; Czechoslovakia; Georgia; Germany; Hungary; Iran; Italy; Kazakhstan; Morocco; Portugal; Romania; Russia; Russia-Rostov Oblast; Russia-Saratov Oblast; Russia-Voronezhskaya Oblast; Slovakia; Spain; Syria; Turkey; Turkmenistan; Ukraine; USSR; Uzbekistan; Yugoslavia (former);

Material examined: Type material. *Schedius flavofasciatus*, Lectotype examined 2 June 2017. circular purple ringed label "LECTO-TYPE"; "Cercedilla C. Bolivar" on reverse "12-viii-16"; "Schedius flavofasciatus Mercet det. J.S. Noyes 1978 LECTOTYPE"; "♀ *Ooencyrtus telenomicida* (Vassiliev) V. Trjapitzin det. 1993"; red label "LECTOTIPO MNCN Tipos N° 10393" "MNCN Ent 183431" (MNCN). Non type material: 20♀, 20♂, ISRAEL, Volcani center, laboratory colony ex eggs of silk moth 2.ii.2015 (Shahar Samra) (initial strain collected in Israel, Eshtaol forest 31°48'N; 35°00'E 250m asl, 26.vi.2011, *Stenozygum coloratum* on caper -Shahar Samra) ISRAEL, 5♀, 10♂, Gilboa, 32°31'N 35°22'E, 200m, 4.viii.2010, Field/Lab reared ex eggs *Stenozygium coloratum* (S. Samra #3); ISRAEL, 25♀, 22♂, Gilboa, 32°31'N 35°22'E, 200m, 4.viii.2010, Orig. field reared ex eggs *Stenozygium coloratum*, Lab. reared ex eggs *Bombyx mori* (S. Samra #9); 270♀, 27♂, FRANCE, SPAIN, CROATIA, ITALY, BULGARIA, GREECE, HUNGARY, ARMENIA, TURKEY, EGYPT, ISRAEL, PAKISTAN ♀♂ (BMNH, DEZA).

Comments: The type of *telenomicida* is missing and our concept of the species is based on material in the BMNH identified authoritatively as such. In general, the species has been separated from closely related ones by the extension of the lineolate sculpture on the scutellum. However, material obtained by rearing the species on a laboratory host (silk moth eggs) shows that this character is extremely variable and is probably unreliable for species discrimination. The species may be highly polyphagous and widely distributed throughout the Palaearctic region and may have been misidentified on several occasions.

**Fig 5:**
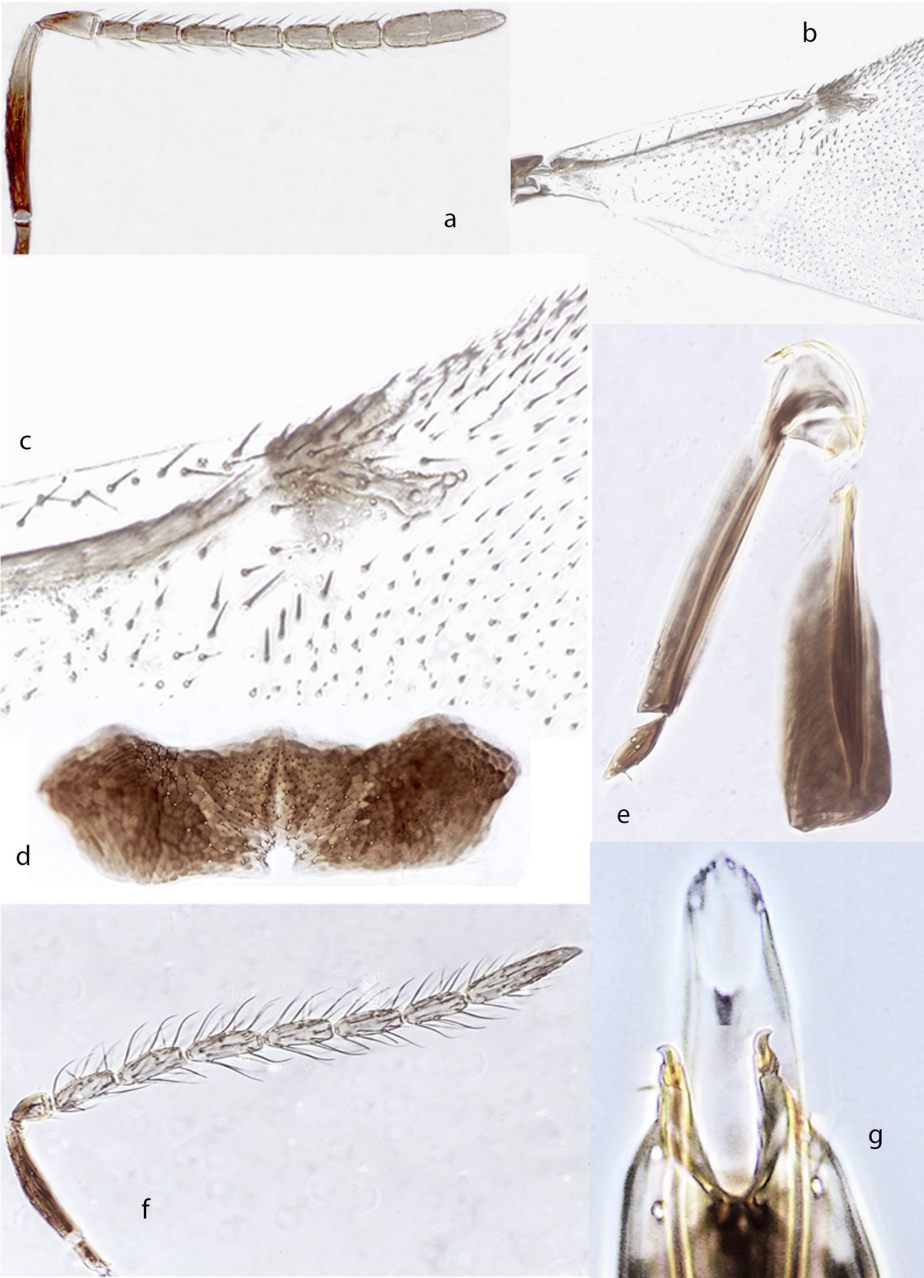
*Ooencyrtus pistaciae* Hayat & Mehrnejad ♀. a. Antenna. b. Fore wing. c. Fore wing venation. d. Hypopygium. e. Ovipositor. ♂. f. Antenna. g. Genitalia.

*Ooencyrtus pistaciae* Hayat & Mehrnejad (Fig 5)

*Ooencyrtus pistaciae* Hayat & Mehrnejad 2016: 200–204. Holotype ♀, Iran, IARI, not examined.

Diagnosis: Female—Head with frontovertex dark brown, with coppery shine; inter-torular area violet; greenish on face and malar space; mouth margin coppery; mesoscutum with faint bluish reflection, a purplish lustre on scutellum and golden green reflection at sides of gaster; antenna (Fig 5A) brown with apex of pedicel paler; legs extensively brown the following parts yellowish or appearing paler: joints and base of all tarsi; distal 2/3^rd^ of mid and hind tibiae; wings hyaline (our specimens have a distinct brown spot below the marginal vein), venation pale brown to brown.

Head 3.5× as broad as frontovertex, with shallow reticulate sculpture made of cell clearly smaller than eye facet; ocelli forming an angle of about 90°; mandible with 2 teeth and a truncation; antenna (Fig 5A) with scape about 7× as long as broad, all funicular segments longer than broad and with linear sensilla except F1; mesoscutum with shallow reticulate sculpture made of irregular cells, clearly different from that of scutellum, coarse, and made of cells elongate and narrow, apical third of scutellum smooth and shiny; fore wing (Fig 5B) 2.2× as long as broad, marginal vein (Fig 5C) about 1.4× as long as broad and as long as postmarginal vein; hypopygium (Fig 5D) transverse, and extending not more than 0.6× the gaster length, its posterior margin with a median invagination; ovipositor (Fig 5E) hidden or hardly exserted, about 0.9× as long as mid tibia or 6× as gonostylus; gonostylus about 0.5× as long as mid basitarsus. Relative measurements: HW 28, FVW 8, POL 4, OOL 1, OCL 2, OD 1, SL 14, SW 2, FWL 66, FWW 29, SMV 24, MV 1.5, PMV 1.5, SV 3; OL 74, MT 85, GL 13

Male: generally similar in appearance to female except for antenna (Fig 5F) and genitalia (Fig 5G).

Hosts. *Stenozygium coloratum*, *Brachynema germarii* (Hemiptera: Pentatomidae) [40]

Distribution. Israel, Iran [40]

Material examined: 20♀, 20♂, Volcani center laboratory colony ex eggs of silk moth 2.ii.2015 (Shahar Samra) (initial strain collected at Israel Ein-Avdat 30°49'N; 34°45'E 500m asl.; ISRAEL, 3♀, 3♂, Beit She'an, 32°28'N 35°31'E, 229m, 15.viii.2013, Orig. field reared ex eggs Stenozygium coloratum, Lab reared ex eggs *Stenozygium coloratum* and *Bombyx mori* S. Samra #4a); ISRAEL, 3♀, 3♂, Lahav, 31°22'N 34°51'E, 480m, 10.vi.2012, Orig. field reared ex eggs *Stenozygium coloratum*, Lab reared ex eggs *Bombyx mori* 24.vi.13 -Shahar Samra (S. Samra #7)) Materal deposited at BMNH, DEZA, TAUI.

Comments: The species has been described and illustrated by the authors [40] in detail. Key characters of the specimens examined here fit with those of description particularly for the colour of the body (including the different metallic shine on different parts of the head), colour and features of antenna (in both sexes), hypopygium and ovipositor.

**Fig 6:**
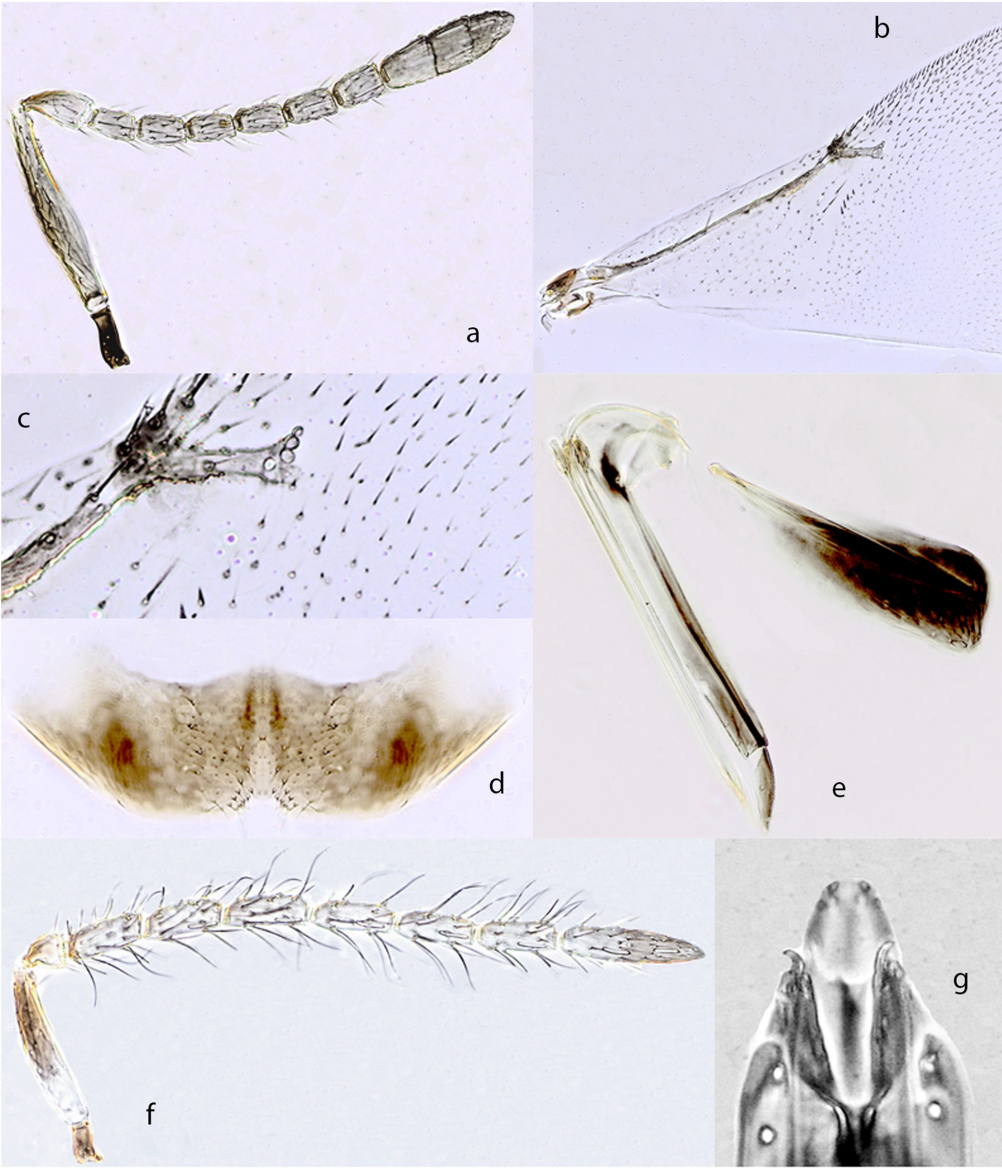
*Ooencyrtus mevalbelus* Guerrieri & Samra sp. nov‥ ♀. a. Antenna. b Fore wing. c. Fore wing venation. d. Hypopygium. e. Ovipositor. ♂. f. Antenna. g. Genitalia.

*Ooencyrtus mevalbelus* Guerrieri & Samra sp. nov. *urn*:*lsid*:*zoobank*.*org*:*act*:*31C0A2F3-8452-490B-8D2E-D10C936E1495* (Fig 6)

Female. Holotype, length 1.1 mm. Head black with very faint metallic lustre on frontovertex, lower part of interantennal prominence with green-purplish reflections; antenna (Fig 6A) light brown, scape yellow with a brown dorsal stripe along the apical 2/3^rd^; mesothorax faintly shiny, scutellum dull with a green lustre at apex and sides; gaster with base yellow, remaining brown; setae on dorsum of thorax whitish; legs yellow except apices of all tarsi, brown; wings hyaline, venation brown; gaster black, the first 2 tergites yellowish;.

Head with shallow reticulate sculpture made of cell clearly smaller than eye facet, 3.7× as broad as frontovertex; ocelli forming an angle of about 90°; mandible with 2 teeth and a truncation; antenna (Fig 6A) with scape about 6.5× as long as broad, all funicular segments longer than broad and with linear sensilla; mesoscutum with shallow reticulate sculpture made of irregular cells, clearly different from that of scutellum, coarse, and made of cells elongate and narrow, apical third of scutellum smooth and shiny; fore wing (Fig 6B) about 2× as long as broad, marginal vein (Fig 6C) about 1× as long as broad and 0.75× as long as postmarginal vein; hypopygium extending not more than 0.6× the gaster length; ovipositor hidden.

Relative measurements: HW 30, FVW 8, POL 4, OOL <1, OCL 2, OD 1, SL 13, SW 2, FWL 65, FWW 31, SMV 24, MV 1.5, PMV 2, SV 4.

Paratype: hypopygium (Fig 6D) about 2.5× as broad as long, its posterior margin with a median invagination; ovipositor (Fig 6E) about 1.1× as long as mid tibia or 5.5× as gonostylus; gonostylus about 0.6× as long as mid basitarsus. Relative measurements: OL 79, MT 70, GL 14

Variation: virtually none in the material at hand except for the body length, which in females varies from 0.9 to 1.2mm.

Male: generally similar in appearance to female except for antenna (Fig 6F) and genitalia (Fig 6G).

Hosts. Reared from eggs of *Stenozygum coloratum* (Hemiptera: Pentatomidae) on caper

Distribution. Israel.

Material examined. Type materal. Holotype ♀, Israel Volcani center laboratory colony ex eggs of silk moth 2.ii.2015 (Shahar Samra) (initial strain collected at Israel Lahav 31°22'N; 34°51'E 480m asl, 10.vi.2012 - Shahar Samra) (TAUI). Paratypes: 2♀, 5♂, same data as holotype (TAUI, BMNH, DEZA). Non-type material: ISRAEL, 4♀, 4♂, Lahav, 31°22'N 34°51'E, 480m, 10.vi.2012, Field/Lab. reared ex eggs *Stenozygium coloratum* (S. Samra #1); 3♀,4♂, Lahav, 31°22'N 34°51'E, 480m, 10.vi.2012, Orig. field reared ex eggs *Stenozygium coloratum*, Lab reared ex eggs *Bombyx mori* (S. Samra # 7)

Comments: See comments under *zoeae*

**Fig 7:**
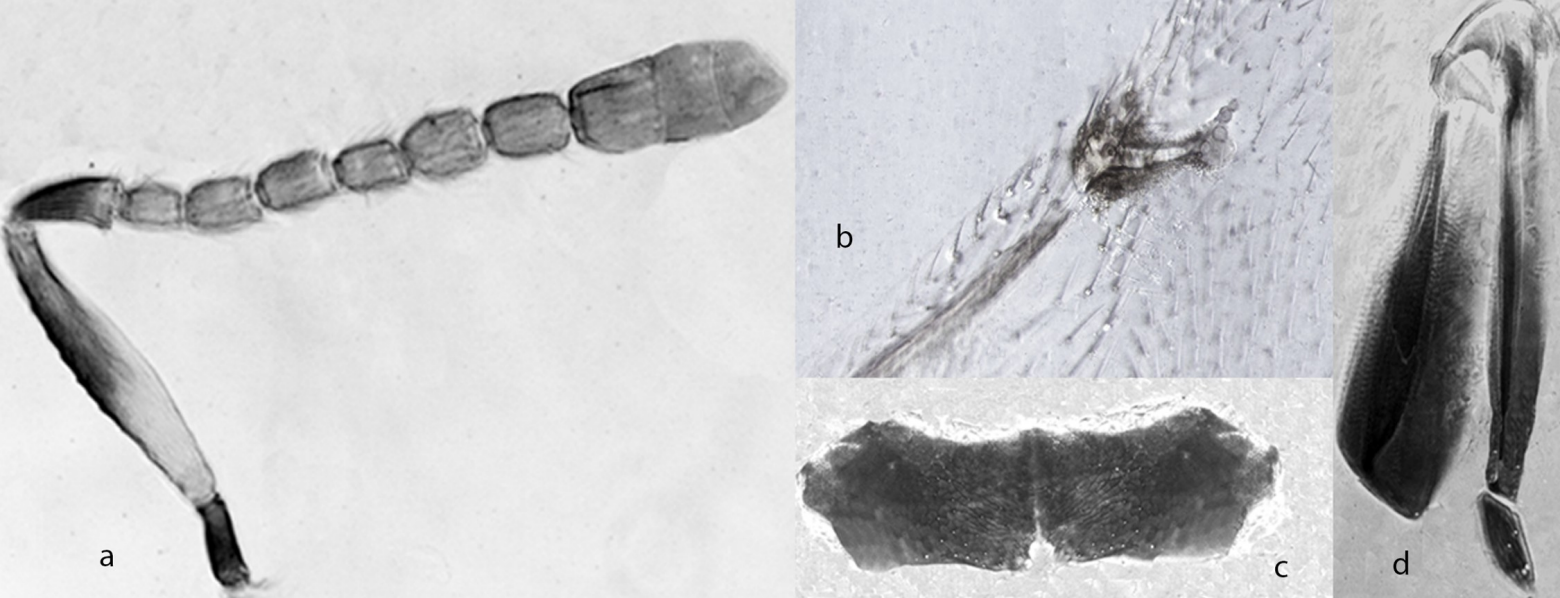
*Ooencyrtus pityocampae* (Mercet) ♀. a. Antenna. b. Fore wing venation. c. Hypopygium. d. Ovipositor.

*Ooencyrtus pityocampae* (Mercet) (Fig 7)

*Schedius pityocampae* Mercet 1921:313. Lectotype ♀, designated Noyes, 1981:183, Spain, MNCN, examined.

*Schedius pityocampae abdominalis* Mercet, 1921:315. Holotype MF, Spain, MNCN, examined. Synonymy with *pityocampae* by Noyes, 1981:183.

*Ooencyrtus pityocampae* (Mercet); Wilkinson, 1925: 9–10.

*Ooencyrtus fecundus* Ferrière & Voegelé 1961: 31-32- Lectotype ♀, here designated, Morocco, MHNG, examined syn.nov.

*Ooencyrtus pityocampae abdominalis* (Mercet); Öncüer, 1991: 207

Diagnosis: Female—Head with frontovertex dark brown, with green lustre; inter-torular area with green shine; mesoscutum and scutellum with green reflection; antenna (Fig 7A) with scape yellowish with a dorsal brow stripe at apical 2/3^rd^, pedicel brown apex paler, remaining segments brown; legs yellow with apex of tarsi and base of hind coxa brown; wings hyaline sometimes with a small brown spot around the marginal vein, venation brown; gaster brown with basal segments paler.

Head with shallow reticulate sculpture made of cell clearly smaller than eye facet, 3× as broad as frontovertex; ocelli forming an angle of about 90°; antenna (Fig 7A) with scape about 7× as long as broad, all funicular segments longer than broad and with linear sensilla except F1; mesoscutum and scutellum with shallow reticulate sculpture made of irregular cells; fore wing 2.3× as long as broad, marginal (Fig 7B) vein about as long as broad and as long as postmarginal vein; hypopygium as in Fig 7C; ovipositor as in Fig 7D. Relative measurements: HW 27, FVW 9, POL 4.5, OOL 1, OCL 1, OD 1, SL 14, SW 2, FWL 60, FWW 26, SMV 24, MV 1.5, PMV 1.5, SV 2.

Male: generally similar in appearance to female except for antenna and genitalia.

Hosts. *Stenozygium coloratum*; *Aelia rostrata* (Hemiptera: Pentatomidae); *Bombyx mori* (Lepidoptera: Bombycidae); *Carpocoris sp*. (Hemiptera: Pentatomidae); *Dendrolimus pini* (Lepidoptera: Lasiocampidae); *Dolycoris sp*., *Dolycoris baccarum*; *Eurydema oleraceum*, *E*. *ventralis* (Hemiptera: Pentatomidae); *Eurygaster maura* (Hemiptera: Scutellaridae)*; Gonocerus juniper* (Hemiptera: Coreidae); *Graphosoma lineatum; Nezara viridula*; *Piezodorus lituratus*; *Rhaphigaster nebulosa* (Hemiptera: Pentatomidae); *Sphinx pinastri* (Lepidoptera: Sphingidae); *Thaumetopoea bonjeani*, *T*. *pityocampa*, *T*. *wilkinsoni*; *Traumatocampa pinivora* (Lepidoptera: Thaumetopoeidae).

Distribution Israel, Albania; Algeria; Bulgaria; Cyprus; France; Greece-main; Italy; Morocco; Poland; Portugal; Spain; Tunisia; Yugoslavia; Yugoslavia (former).

Material examined. Type material, *Schedius pityocampae*, Lectotype ♀, SPAIN, "Guadalajara" "ex huevos de procesionaria del pino" (MNCN); Paralectotype, 1♀, "SPAIN, Guadalajara, 1922.168", "*Schedius pityocampae* Mercet Parasito de Thaumet. *pityocampa cotipo*" "*Ooencyrtus pityocampae* Mercet, det J.S. Noyes 1979" "*Ooencyrtus pityocampae* Mercet det J.S. Noyes, 2014" "compared with lectotype 9.xii.2014" (BMNH); *Schedius pityocampae abdominalis*, Holotype ♀, "Madrid, 9-IX-1917, Pinus" "*S*. *pityocampae* var fasciatus" (MNCN) *Ooencyrtus fecundus*: LECTOTYPE ♀, (here designated, remounted by JSN from minuten pin onto a minuten pin on card point above card rectangle), "*Ooencyrtus Aeliae* ♀" "COTYPE" "Select as primary type *Ooencyrtus fecundus* ♀. + V. det J.S. Noyes 2015"; PARALECTOTYPES, 9 ♀ TYPESECMF "Maroc, iii.59 Mekhés Voegelé Ex oeufs de Aelia" "Encyrtidae: *Ooencyrtus fecundus* n.sp. ♀ [in pencil] Ch. Ferriére det."; 11 ♀,14♂ (on 1 slide, with heads mostly separated) "Maroc, iii.59 Mekhés Voegelé Ex oeufs de Aelia B [in pencil]" "Encyrtidae: *Ooencyrtus fecundus* n.sp. Ch. Ferriére det."; 5♂ (on slide) "Maroc, iii.59 Mekhés Voegelé Ex oeufs de Aelia" "Encyrtidae: *Ooencyrtus fecundus* n.sp. ♂ Ch. Ferriére det."; 5♀ (dismembered on 1 slide) "Maroc, iii.59 Mekhés Voegelé Ex oeufs de Aelia" "Encyrtidae: *Ooencyrtus fecundus* n.sp. B[in pencil] Ch. Ferriére det."; 9♀, 6♂ (on minuten pins, only 1♀ 2♂ intact, remainder mostly lost head or other body parts; the female specimen treated similarly to lectotype and is in good condition but the head has slightly collapsed), "*Ooencyrtus Aeliae* ♀" "COTYPE"; 5♂ (on card points), "Maroc Mekhés 1960 Voegele" "TYPUS". Non type material. ISRAEL, 2♀, 2 ♂, ISRAEL, Gilboa 32°31N; 35°22'E 200m asl, 4.viii.2010 (Shahar Samra) (deposited at TAUI); 331♀, 10♂, FRANCE, SPAIN, PORTUGAL, TURKEY, CYPRUS, ITALY, BULGARIA, CROATIA, GREECE, ISRAEL, MOROCCO (BMNH); 15♀, 15♂, ITALY (DEZA).

Comments: The species is one of the easiest to recognize amongst palaearctic ones because of the distinctive colour/sculpture of mesoscutum and scutellum coupled with yellow legs. According to Ferriére & Voegelé the holotype, allotype and 20 paratype males and females of *O*. *fecundus* were deposited in MHNG with a further 20 paratypes (♂♂and ♀♀) deposited in Direction de la Recherche Agronomique in Rabat, Morocco. In the type series there is no indication of the holotype but there are four males labelled as "TYPUS". None of them could be the holotype because the authors explicitly stated that the holotype is female. We then assume that the authors just forgot to properly label the type material and thus, according to ICZN rules, designate here a Lectotype of *O*. *fecundus*. We have examined the type series of *O*.*fecundus* and have no doubt in synonymize it in *pityocampae*.

## Discussion

The present study focuses on the *Ooencyrtus* spp. parasitoid complex occurring in the CB eggs. A major challenge in identification of these species arose from the great lack of distinguishable and reliable morphological differences, coupled with high morphological polymorphism. Therefore, a molecular approach was essential. It is likely that without the use of molecular screening, the presence of at least one of the species, *O*. *zoeae*, would have passed unnoticed. Generally, the molecular approach enabled a clear separation between the congeners, which was also supported by the crossbreeding trials. Although *O*. *pityocampae* and *O*. *pistaciae* were not included in these trials, clear genetic separation suggests that they, also, are reproductively isolated from the rest. Furthermore, two species that were tested in the crossbreeding trials and could not crossbreed—*O*. *mevalbelus* and *O*. *zoeae—*were found to be the most closely related, which suggests that the other congeners, which are more genetically and morphologically distinct, are even less likely to crossbreed.

Nonetheless, further steps are needed in order to complete the identification process and determine phylogenic relationships. Several problems remain to be resolved. First, two distinct COI haplotypes of *O*. *pistaciae* were obtained, which raises the question of whether these two clades represent a single species or two separate ones. Furthermore, although the studied species were well separated according to both DNA fragments, it was difficult to determine their phylogenetic relationships, because of the similar distances separating some of the species, and the inconsistency between the relative genetic distances obtained from the respective ITS2 and COI analyses. Consequently, the relationships between species cannot be fully determined. Interesting findings were obtained by phylogenetic analysis with a slightly larger COI fragment of 1237 bases—which included the presently used 946-bp fragment—that was obtained with LCO1490 and the reverse primer used in the present study. These results show a more similar picture to that obtained with the ITS2 (Samra, unpubl. data), in which *O*. *pityocampae* was placed outside the grouping of the other four species, thus supporting the hypothesis of an earlier divergence of *O*. *pityocampae* from the others. However, the presence of pseudogenes prevented sequencing of *O*. *zoeae* with this primer set, and a poly-t region near the 5' end of this DNA segment caused considerable difficulty in obtaining clear sequences also for the other species. Thus, only a few sequences were obtained from four species and, therefore, we were obliged to use the shorter 946-bp fragment.

In Israel of the *Ooencyrtus* spp. obtained from CB eggs, *O*. *pityocampae* is the only uniparental species. Its thelytoky is almost certainly induced by *Wolbachia* [41]. This endosymbiont bacterium is known to interfere with the reproductive mode of many insect species, and to induce thelytoky in other hymenopteran parasitoids [42,43]. There is evidence that it also may cause a reduction in mitochondrial haplotype diversity through a process of selective sweep [44]. Therefore, *Wolbachia* also may account for the low genetic diversity observed in COI haplotypes of *O*. *pityocampae*. However, both Israeli and Turkish *O*. *pityocampae* populations are infected with the same *Wolbachia* strain (Samra, unpubl. data), and it is not clear why the Israeli population is much less diverse than the Turkish one. Most probably, uniparental populations of *O*. *pityocampae* from Morocco are similarly infected [16,45]. Therefore, the exceptionally low genetic diversity in the Israeli *O*. *pityocampae* population is probably associated with a different process; possibly it results from a relatively recent expansion of this species, following the arrival and spread of the PPM in Israel [46]. However, biparental poplations of *O*. *pityocampae* have been collected and it would be worthy to characterize them to unravel their relation with uniparental ones. In contrast, the level of genetic diversity of *O*. *telenomicida* and *O*. *zoeae* is similarly high in both Turkish and Israeli populations, which indicates that the situation in *O*. *pityocampae* can be considered unique.

Some of the *Ooencyrtus* spp. identified in the present study are known natural enemies of various Lepidopteran and Heteropteran pests, e.g., *Nezara viridula* and *Aelia* spp. (Heteroptera: Pentatomidae) and PPM [14,16,17]. Their frequent occurrences on CB eggs suggest that this bug might be important for conservation of these egg-parasitoid populations in the east Mediterranean area. Although the CB is known to attack various agricultural plant species [47], it is not considered a significant pest, because damage to agricultural crops is usually quite rare and localized [30,47]. In fact, CB individuals probably cannot reproduce on plants other than capers [30], therefore, switching to other plants is most likely to occur only in late summer when capers' vital foliage becomes scarce. Hence, we suggest that enhancing CB populations may lead to an increase in the populations of its egg parasitoids, which, in turn, might help to control the populations of more significant pest species that occur in the same area. This seems especially relevant to the case of *O*. *pityocampae* and the PPM. The eggs of the latter species are laid mainly in September–November [14], therefore, the *O*. *pityocampae* population most likely relies on alternative hosts to survive through the spring and summer months (April–August) which coincide closely with the reproductive period of CB [31]. Host switching may also be essential for survival of the other discussed *Ooencyrtus* spp., though, they do not parasitize PPM eggs (Samra unpubl. data).

Finally, we suggest that the molecular data on the various *Ooencyrtus* species gathered in the present work could help to elucidate the identities of other *Ooencyrtus* populations, found on various other hosts in the area. Accurate identification is vital to enable validation of their true distributions and host ranges, and evaluation of their role as mortality agents of their hosts.

## Supporting information

S1 Appendix List of *Ooencyrtus* species from *Stenozygum coloratum* eggs which were misidentified in previous publications. (DOCX)
