# Supplementary material for: Diversity of Ooencyrtus spp. (Hymenoptera: Encyrtidae) parasitizing the eggs of Stenozygum coloratum (Klug) (Hemiptera: Pentatomidae) with description of two new species
Source: PLoS One. 2018 Nov 7;13(11):e0205245. doi: 10.1371/journal.pone.0205245 (PMC6221264; doi:10.1371/journal.pone.0205245)
Supplement: S1 Appendix — (DOCX) [file pone.0205245.s001.docx]

**S1 Appendix. List of *Ooencyrtus* species from *Stenozygum coloratum* eggs which were misidentified in previous publications.**

| Species | Misidentified as |
| --- | --- |
| *Ooencyrtus mevalbelus* | *O. fecundus*^1,2,3^ |
| *O. pistaciae* | *O.* near *nigerrimus*^2^, *O. nigerrimus*^3^ |
| *O. zoeae* | *O.* near *fecundus*^1,2^ |

^1^ Samra S, Ghanim M, Protasov A, Mendel, Z. Comparative study of development parameters of four *Ooencyrtus* spp. (Hymenoptera: Encyrtidae) on natural and factitious hosts. J. Appl. Entomol., 2015; 140: 334-345.doi:[10.1111/jen.12264](https://doi.org/10.1111/jen.12264)

^2^ Samra S, Ghanim M, Protasov A, Mendel Z. Spatial distribution and niche partitioning in the *Ooencyrtus* spp. complex parasitizing the eggs of *Stenozygum coloratum*. BioControl 2015; 60: 747-760.doi.org/10.1007/s10526-015-9683-2

^3^ Samra S, Murad G, Protasov A, Mendel Z. Seasonal history, plant interactions and egg parasitism of the variegated caper bug, *Stenozygum coloratum* Klug (Heteroptera: Pentatomidae). Agricultural and Forest Entomology 2016; 18(1): 22‐34. doi.org/10.1111/afe.12123
